# Participation Strategies Used by Young People With and Without Physical Disabilities

**DOI:** 10.1177/15394492241280198

**Published:** 2024-09-24

**Authors:** Andrianantenaina Ornella, Baladzhanov Nikol, Germain Lauriane, Schneidman Lyndsay, Shahin Saeideh, Anaby Dana

**Affiliations:** 1 McGill University, Montreal, Quebec, Canada; 2 Centre de Recherche Interdisciplinaire en Réadaptation de Montréal Métropolitain (CRIR), Montreal, Quebec, Canada

**Keywords:** participation strategies, transition-age youth, physical disability, environment

## Abstract

Participation strategies used by young people (YP) are understudied. This study aimed to identify strategies used by YP with and without physical disabilities to promote participation at home, school, community, and workplace. In this qualitative descriptive study, 106 participants ages 12 to 30 ( $\bar{x}$ = 22.7) years, with (*n* = 52) and without (*n* = 54) physical disabilities, reported up to three strategies to facilitate their participation in four settings using the Youth, Young-Adult Participation and Environment Measure. A total of 1,235 strategies were analyzed using inductive content analysis. As a result, 11 categories emerged; seven were environment-focused and four were person-focused. Both groups used time management, built on intrinsic factors, and utilized social support across all settings. At school, those with disabilities uniquely benefited from accommodative institutional environments. They also reported more strategies involving advocacy, analyzing a situation, and seeking accommodative physical and social environments. The findings illustrate a range of participation strategies YP use that may inform ways to promote participation more effectively.

## Plain Language Summary

What Strategies Did Young People With and Without Physical Disabilities Use to Facilitate Their Participation During the COVID-19 Pandemic?

This study describes self-reported strategies used by 106 young people with and without physical disabilities to promote their participation in a range of activities for youth aged between 12 and 30 years who are transitioning to adult roles. Seven out of eleven strategies identified focused on modifying the environment. While both groups used common strategies (e.g., time management, social support) across the home, school, community, and workplace settings, those with disabilities reported unique strategies related to school participation, such as requesting accommodations and seeking recourses. More strategies related to advocacy, analyzing situations (e.g., modifying activities), and benefiting from accommodating physical and social environments were seen among those with disabilities. The findings can enhance our understanding of participation strategies used by young people and the gaps that exist. Together, clinicians and youth can build on this knowledge to promote participation and inclusion in a client- and family-centered practice, using solution-based intervention strategies.

## Introduction

Participation, defined as “involvement in a life situation,” is determined by a combination of personal and contextual factors (Law, 2002), and is positively associated with well-being (Augustine et al., 2022). Personal factors, such as unique sets of motives, patterns of organization, abilities, and limitations, as well as contextual factors including the environment can either foster or restrict participation (Killeen et al., 2019). The COVID-19 pandemic is another factor that significantly affected participation at home, school, community, and the workplace. This unprecedented event—mandating households to stay indoors and adhere to social distancing regulations in various locations—led to sudden and significant alterations in daily engagement across settings (Wang et al., 2024).

Compared with their typically developing peers, young people with physical disabilities have limited participation across various domains, such as social life and interpersonal relationships (Guyard et al., 2024), spending less time with friends and in sports (Michelsen et al., 2014). Participation limitations, especially during the complex transition to adulthood, translate into challenges in acquiring desired adult roles, notably in major life areas, such as education, employment, community life, and relationships (van Gorp et al., 2019). Similarly, in a qualitative study, young adults with cerebral palsy (CP) describe how “branching out into adulthood” incorporated participation challenges (Ding et al., 2024). There is an emerging body of knowledge exploring types of strategies specifically used by parents or caregivers to support the participation, in home and community activities, of young children receiving early intervention (Kaelin et al., 2021) at school (Kaelin et al., 2023) and across various settings, among youth ages 12 to 18 years living with a physical disability (Killeen et al., 2019). Examples of parental strategies included modifying the environment and incorporating the child’s interests to bolster their involvement (Kaelin et al., 2021; Killeen et al., 2019).

Although such knowledge provides valuable insight, there is a gap in research regarding strategies identified and used by older youth with disabilities regarding their own participation across a broader range of settings, including the workplace. Thus, this study aimed to explore the types of strategies young people with and without physical disabilities, ages 12 to 30 years, used to facilitate their participation at home, school/educational setting, community, and the workplace. Furthermore, similarities and differences in the type and scope of participation strategies were explored. Generating such knowledge is imperative in advancing our understanding of the range of strategies used to further promote participation and inclusion in meaningful occupations among young people, especially during the COVID-19 pandemic that posed greater participation challenges.

## Method

This qualitative descriptive study identified and described young people’s self-reported participation strategies collected as part of a study investigating the initial psychometric properties of the Youth, Young-Adult Participation and Environment Measure (Y-PEM; Shahin, Ahmed, et al., 2023). Specifically, it uniquely analyzed open-ended responses about strategies youth provided on the Y-PEM.

### Participants

Data from 113 participants with (*n* = 56) and without physical disabilities (*n* = 57) were collected. Participants were included if they were aged between 12 and 30 years, and able to read and understand English or French at Grade 5 level. Participants with physical disabilities were included if they had a mobility restriction based on therapist’s input or self-reported information. Those with cognitive impairments and intellectual disabilities were excluded based on input from a therapist who was independent of the study. Participants with physical disabilities were recruited using convenience sampling through local coordinators from nine programs in three Quebec-based rehabilitation centers and through ads posted in disability advocacy groups on social media (i.e., Twitter, Facebook). Other recruitment strategies included contacting six community-based employment services. Participants without physical disabilities were mainly recruited from Quebec, using the research team’s personal network with purposeful sampling to ensure that both groups were proportionally similar in terms of sex and age. Informed consent was obtained from all participants through Research Electronic Data Capture (Harris et al., 2009).

### Procedures

Consenting participants completed the Y-PEM and a demographic questionnaire in English or French, based on their preferred language, on REDcap. All data were collected during the COVID-19 pandemic, from January 2020 to October 2021.

### Assessments

#### The Y-PEM

The Y-PEM is a self-reported measure that evaluates participation in 31 sets of activities across four settings (home, school/educational, community, and the workplace) and the impact of the environment on participation in each setting (Shahin, Ahmed, et al., 2023). At the end of each setting, young individuals opt to report, in their own words (with no word limit), up to three strategies they use to promote their participation, resulting in up to 12 strategies (3 [strategies] × 4 [settings]) per participant. Content validity of the Y-PEM was supported among young people with and without physical disabilities (Shahin et al., 2022) and this measure has demonstrated acceptable to good internal consistency (.71–.82) and test–retest reliability (.70–.85) across 10 out of the 12 scales (Shahin, Ahmed, et al., 2023). It has also been perceived as a valuable tool with relatively low burden by young people with visual impairment (Ryan et al., 2023) and its utility was supported by stakeholders in the field of employment (Shahin, Ryan, et al., 2023).

#### Demographic Questionnaire

A demographic questionnaire was used to collect data regarding participants’ personal factors, such as age, sex, language, level of education, vocational status, and living situation. In addition, participants with physical disabilities reported their functional challenges (FCs) using a study-specific checklist of 11 areas of function (no problem [0], little problem [1], and big problem [2]). The FCs included motor (moving around, using hands to do activities), affective (managing emotions), cognitive (paying attention), learning (e.g., remembering information), and sensory components (seeing, hearing), among others. A FC was attributed to each area with a score of 1 or 2. The number of FCs reported ranged from 0 to 11. The frequency of each FC was calculated and presented in percentages. This checklist has been successfully used in previous studies examining levels of participation (Anaby et al., 2014).

### Data Analysis

Demographic information was analyzed using descriptive statistics. An inductive content analysis of the reported strategies was conducted following Elo and Kyngäs’s (2008) process using Microsoft Excel. A unit of analysis (i.e., few words) was selected in preparation for coding. The first 10% of narrative responses were pilot-coded by all team members to establish a common coding scheme. Then, two team members (A1 and A3) independently coded the strategies across all settings in three rounds and generated an initial list of codes per group (with and without disability). Discrepancies were resolved through discussion with a third team member (A2). Coding discrepancies pertained to whether a response qualified as a strategy, whether a response included multiple strategies, and whether the attributed code was representative of the response. Responses that alluded to inapplicability (e.g., “not applicable”) and duplicate answers within one participant’s response were not considered in the analysis. Responses were decoupled and coded separately when they included multiple strategies (e.g., “adjustable office supplies, listen to music while working”). Subsequently, two different team members (A2 and A4) sorted the codes into subcategories, then collapsed them into generic categories and finally into main categories. All team members participated in four meetings to discuss the categorization process that was validated by two experienced data analysts (A5, A6). Discrepancies in this process were also resolved through discussion with the research team. Generic and main categories were generated for the two groups (i.e., young people with and without physical disabilities) in each of the four settings.

The number of strategies (in English and French) that supported the generic and main categories were counted across all four settings and within each of the settings to describe the distribution of strategies across the two study groups. Bar charts were used to display and highlight the differences and similarities in frequency of these categories, presented in percentages.

## Results

### Sample Characteristics

Strategies from 106 participants (*n* = 52 with disabilities; *n* = 54 without disabilities) ages 12 to 30 years (*x−* = 22.7) were included in the analysis. Seven participants were excluded from the analysis due to missing data. A total of 89 participants reported strategies in English and 17 in French. As shown in Supplemental Table S1, participants with and without physical disabilities were proportionally similar in terms of sex, age, type of community they lived in, and their occupation (e.g., working, studying). About 40% of participants with physical disabilities had a high school degree or less and 58% were involved in work-related activities (full-/part-time/volunteering). About 44% of young participants without physical disabilities had graduated from college or university, and 85% were involved in work-related activities.

Participants with disabilities reported up to 10 FCs, with 50% of them having more than five FCs. The most frequent FCs were difficulties in moving around (80%), using hands to do activities (69%), paying attention or concentrating (67%), reacting to sensations (53%), managing emotions (53%), and remembering information (53%).

### Reported Strategies

Overall, 1,017 data entries resulted in 1,235 available strategies for coding (as some entries encompassed more than one strategy). Of them, 347 strategies (28%) were reported at the home, 302 strategies (25%) in the school/educational setting, 312 strategies (25%) in the community, and 274 strategies (22%) in the workplace.

Overall, 11 main categories of strategies were identified in both groups. Of these 11 main categories, seven categories were associated with the participants’ environment: cultivating an optimal physical environment (e.g., access to technology and home adaptations), receiving social support (e.g., connection with peers and family), benefiting from an accessible school physical environment (e.g., accessing educational aids), an accommodative learning environment (e.g., academic accommodations), availability of services and resources in the educational setting (e.g., access to support services, such as a disability counselor or special education instructor), and benefiting from an accommodative work environment (e.g., access to flexible working conditions) and accessible work physical environment (e.g., access to equipment or transportation). The remaining four main categories of strategies were the following: implementing time management techniques (e.g., planning ahead and establishing a routine), building on intrinsic factors (e.g., nurturing motivation and self-reliance), analyzing the situation (e.g., prioritizing tasks or maintaining an optimal pace within specific contexts), and optimizing resource utilization (e.g., seeking for services and activities). Table 1 illustrates the definition of each category of strategies, the setting where it was used, and corresponding examples.

**Table 1.** Examples of the Range of Distinct Strategies Used by Young Adults With and Without Physical Disability.

Main categories	Settings	Definitions	Examples of strategies	
			With disabilities	Without disabilities
Implementing time management methods	Home, school/educational setting, community, and workplace	Application of diverse strategies, aids, and techniques to effectively plan, organize, and allocate time for a range of tasks and activities.	I use my agenda to plan activities around workdays and rest periods before and after work (work setting).	I use an agenda and calendar to keep track of assignments (school setting).
Receiving social support		Various forms of interaction within one’s social circle, including help, assistance, advice, engagement in shared activities, and communication with family members, friends, peers, teachers, and mentors.	My parents assist me with self-care I am unable to complete on my own (e.g., clipping nails, cleaning ears; home setting).	Ask peers and teachers for support when needed (school setting).
Utilizing intrinsic factors or qualities		Use of inherent traits, strengths, and	I seek and request accessible services (school setting).	I try to engage in learning-type activities at work and learn from the experience of my coworkers (work setting).
		abilities, such as self-reliance, choice-making, advocacy initiatives, and motivation, to improve overall well-being, effectiveness, and success in activities.		
Analyzing the situation	Home, community, and workplace	Assessing task demands, understanding capabilities, the context of the activity, and informed decision-making to optimize participation.	It’s a lot of planning in advance, but we try and do less demanding things on days where the weather is “bad” (humid, very cold, or too warm) as that type of weather irritates my joints, bones, and articulations more (community setting).	With the ongoing pandemic, there has been very limited community involvement. We still try to keep the same activities ongoing adapted to the current circumstances (community setting).
Cultivating an optimal physical environment	Home, community	Intentional actions, strategies, and equipment to overcome obstacles that could hinder participation and modification of the physical layout and features of a space to ensure optimal access and usability, tailored to the individual’s specific needs.	I wear my back brace before getting into chores or if I must stand for a long time (home setting).	I dedicate a space for work-/school-related activity (home setting).
An accommodative environment	Workplace	Broad range of strategies and policies (e.g., flexible working conditions) to establish an inclusive workplace culture that accommodates and supports the needs of employees.	My superiors are very accommodating to my arthritis as I work in a children’s clinic (they are aware of the health conditions). They are sometimes the ones to propose and find new alternatives for me and for my whole department to adopt. For example, having everything numerized electronically and using a computer for the majority of the time.	I have flexible hours in my work allowing me to work when I want and how I want.
An accessible physical environment		Provision of technology, equipment, accessible means of transportation, and the restructuring of the workplace’s physical layout, so that individuals can effectively engage in their work tasks.	Workplace adaptation: automatic door, desk height.	The material or equipment I need is provided for me (such as uniforms and meals).
An accessible physical environment	School/educational setting	Intentional design, layout, and features of physical spaces within an educational institution, the accessible transportation options that are intentionally structured and equipped to cater to the needs and accessibility requirements of students.	Having access to the disabled transport shuttle was amazing for getting around campus that was full of hills and long walks . . . but most of the time, it wasn’t available, and/or it made me up to 20 min late for my classes.	There are many lounges, libraries, study areas, and so forth, in the school that gives us a chance to get together and hang out with peers.
An accommodative learning environment		Tailored educational setting that is intentionally structured to provide academic accommodations and enable access to the preferred mode of educational delivery that meet and support the learning needs of students.	I had many accommodations for gym, where I wasn’t necessarily graded on how much I do during class like the others but on how much I participate and include myself in activities (the quality of what I do instead of quantity). My gym teacher also included some of my physio exercises into our stretches and warm-ups. So that was amazing because it made me feel normal and included.	Requested extra time for my assignments and tests.
Available services and resources		Academic supports, facilities, personnel, programs, and extracurricular activities used to enhance student’s school experience and well-being, and facilitate their academic progress.	I meet with my disability counselor at the beginning of each year and as needed.	I use other notetakers’ notes.
Making use of resources and available information	Community	The active engagement of individuals in seeking and leveraging services, and information available within their local community to stay updated on events and enhance social interactions and participation in activities.	I sign up for local newsletters to stay updated on events and activities.	Reaching out to the community center and asking about the activities offered and their schedule.

The most frequently reported main category across settings and groups was implementing time management methods (32%). Specifically, in the home setting, implementing time management methods appeared in 147 out of 347 strategies (42%) in both groups combined. In the educational setting, most strategies fell under the main category of benefiting from an accommodative learning environment (24%) for young people with disabilities, as opposed to implementing organization and learning techniques (31%) for participants without disabilities. In the workplace, the most frequent categories reported by people with disabilities were utilizing intrinsic factors or qualities (23%) and implementing organization and productivity techniques (22%). For participants without disabilities, the predominant strategy was only the latter (44%). In the community setting, young people with disabilities reported more strategies related to cultivating an optimal physical environment (24%), whereas social support (28%) was the most common category for young individuals without disabilities.

#### Similarities Among Groups and Settings

**Figure 1:**
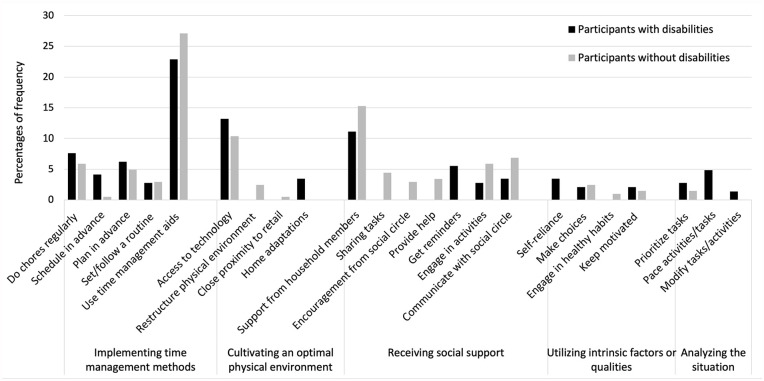
Distribution of Strategies at the Home Setting (n = 347).

**Figure 2.**
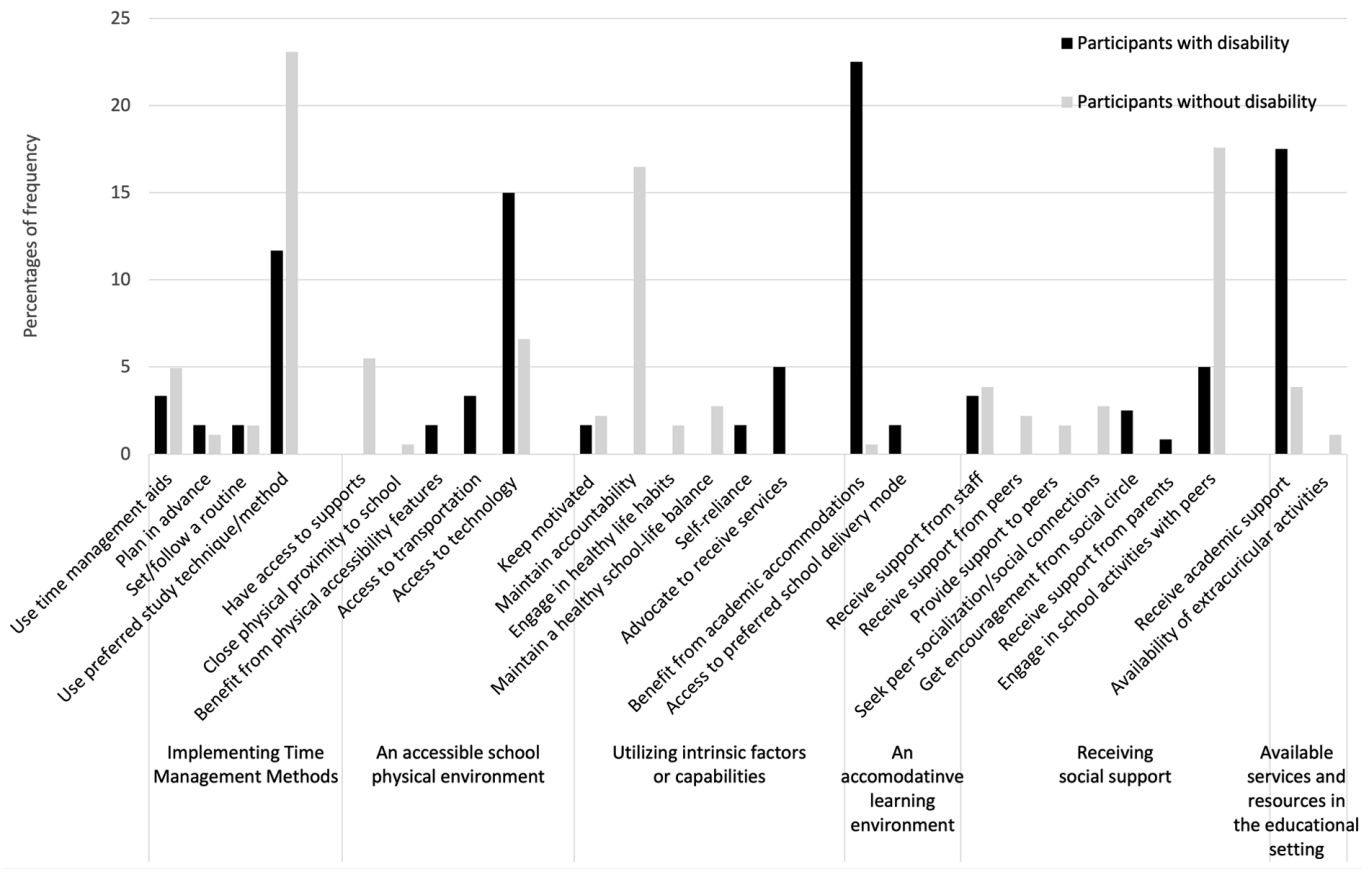
Distribution of Strategies at the School/Educational Setting (n = 302).

**Figure 3.**
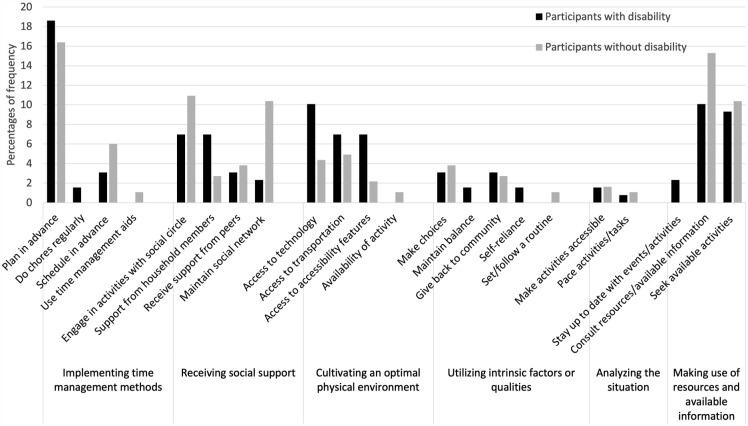
Distribution of Strategies at the Community Setting (n = 312).

**Figure 4.**
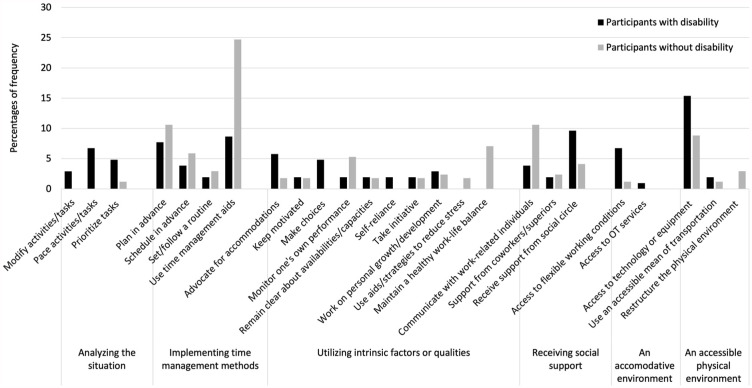
Distribution of Strategies at the Workplace Setting (n = 274).

Four to six main categories of strategies were identified in each setting. Figures 1 to 4 illustrate the main categories and the frequency of the underlying generic categories pertaining to the home, school/educational setting, community, and the workplace, respectively. Three categories of strategies, that is, implementing time management methods, receiving social support, and utilizing intrinsic factors or qualities were identified in both groups across all the settings. In the home setting, young people with and without disabilities used “implementing time management methods,” such as calendars and agendas for scheduling and task tracking, to participate in household chores or activities. In the school/educational setting and the workplace, study/work methods, such as note-taking, reviewing recordings, and using time management aids/tools, served as strategies for participation. In the community, both groups highlighted the importance of planning transportation and organizing activities in advance, completing other tasks prior to engagements, and coordinating schedules with social circle.

Across all settings, participants from both groups benefited from “receiving social support” through communication with family members and asking for help when needed. In the school/educational setting, staff support and connection with peers were key to enable participation in educational tasks and extracurricular activities. In the workplace, transparent communication with colleagues, supervisors, and clients was highlighted and involved seeking assistance, attending meetings, delegations, and nurturing connections. In the community, both groups reported engaging with peers to support their participation. Encouragement played a pivotal role for both; however, participants without disabilities also emphasized mutual help by offering to plan out community activities.

“Utilizing intrinsic factors or qualities” was another category of strategy used by both groups across all settings to foster motivation (e.g., listening to music, rewarding themselves after a task). Other strategies related to taking initiative (e.g., suggesting solutions for improved work efficiency), making choices (e.g., choosing community activities based on interests), and advocating for oneself (e.g., work accommodations) fell under this category.

The environment was identified to be an important consideration for optimizing participation among both groups across all settings. The generic category of “access to technology such as smartphones or laptops to get organized” and “social media to stay connected” was integral in facilitating participation. The availability of resources, such as transportation services, parking facilities, and availability of activities, was also identified as affecting participation. Notably, participants with disabilities descriptively provided a greater number of strategies under this category. Examples include using adapted equipment (e.g., reserving wheelchairs in attraction parks), seeking accessible activities through community channels, and inquiring about accessible features in the community (e.g., checking the availability of reserved sections for people with disabilities at shows).

#### Differences Among Groups and Settings

##### The School/Educational Setting Displayed the Biggest Differences Between the Two Groups

Based on Figure 2, notable disparities between the two groups are most prominently observed in strategies used within the school/educational setting. Specifically, a larger proportion of participants with disabilities discussed strategies falling under generic categories of access to technology (e.g., using a laptop, iPad, and voice-to-text software), utilization of academic accommodations (e.g., being granted extra time to complete examinations or submit a paper, writing exams on a laptop instead of handwriting), and availing academic support (e.g., tutoring, specialized teaching, and utilizing the school’s accessibility center).

A greater proportion of participants without disabilities shared strategies related to using a preferred method for studying, but shared similar study methods (e.g., taking notes, reading aloud, and preview lecture material before class). Unique to those without disabilities was the subcategory of “maintaining accountability” (e.g., attending to tutoring or study sessions). This group was also significantly more engaged in school activities with peers, participating approximately five times more than young people with disabilities. These collaborative activities included studying or participating in extracurricular activities with friends, such as class groups, sports clubs, and study groups.

##### Participants With Disabilities Mentioned More Strategies Related to Advocacy Within Utilizing Intrinsic Factors

One of the distinct intrinsic factors utilized by young people with disabilities was advocacy, particularly in the school/educational and workplace settings. However, it was infrequently reported (5%–6%). Participants with disabilities exhibited advocacy by actively pursuing accessible services and disability-related programs, and through strategies, such as requesting extensions for paper submissions, seeking additional support in challenging situations, and expressing the need for remote work or accommodations.

##### Participants With Disabilities Mentioned More Strategies Related to Analyzing a Situation

In three settings (home, community, and workplace), although with a relatively low frequency (up to 14%), participants with disabilities exhibited more strategies pertaining to “analyzing the situation” and considered a wider range of factors when assessing an activity demand or context. These involved pacing, adapting, or modifying the task or activity, or considering additional factors, such as energy level, presence of accessibility features, or weather conditions. They mentioned the need to organize for rest intervals or transport accordingly. For example, in the community, participants with disabilities evaluated the accessibility of an activity and planned accordingly. Some mentioned having to check the weather conditions, the availability of reserved parking spot, or whether a building is wheelchair friendly.

##### Participants With Disabilities Mentioned More Strategies Related to Benefiting From an Accommodative Environment

Another main difference between strategies used by young people with and without disabilities was seen in strategies relating to the accommodating physical, social, and institutional environment. For instance, regarding the physical environment at home, participants with disabilities emphasized the value of home adaptations such as elevating platforms or floor-level showers. Outside the home, they also emphasized the value of physical accessibility features that included elevators, automatic doors, and optimal space layouts. Moreover, young individuals with disabilities distinctively reported receiving reminders at home and encouragement from their social circles at school, highlighting the supportive role of friends and family in facilitating their participation. In the school/educational setting, aspects of the institutional environment with accommodations, such as online exams, extended test time, early class departure, prolonged assignment deadlines, and accessibility centers, were identified. Specialized resources, including Education Resource Teachers, disability counselors, helpers, and psychologists for support, were also used to facilitate participation. In the community, accessible features, such as transportation, parking, school buses, or adapted transport, were mentioned to be imperative for optimal participation. At work, participants with disabilities specifically mentioned technologies, such as voice-to-text software, digitized documents, or online meetings.

### Discussion

This study reveals 11 types of strategies used and reported by young people with and without physical disabilities to facilitate their participation in the home, school/educational setting, community, and the workplace. Study findings stipulate that, in their transition to adulthood, young people adopted a broad range of strategies with a special focus on the environment, representing seven out of 11 categories of strategies identified. Similarly, the environment has been emphasized as a focus in prior studies examining the strategies used by parents or caregivers to promote the participation of children and youth with physical disabilities (Killeen et al., 2019; Piskur et al., 2012), children receiving early intervention (Kaelin et al., 2021), and children surviving critical illness (Jarvis et al., 2018). Importantly, young people with physical disabilities based their strategies on various facets of the environment, including physical aspects, by “cultivating an optimal physical environment”; social, through “receiving social support”; and institutional, alluding to “available services and resources.” This coincides with previous scoping reviews that found that all environmental domains of the International Classification of Functioning, Disability, and Health (ICF) had an influence on children and youth with disabilities’ out-of-school participation (Anaby et al., 2013) and on transition-age youth with brain-based disabilities’ workplace participation (Shahin et al., 2020). Interestingly, the social aspect of the environment was recurrent in all settings for both groups. This finding is in line with a prior qualitative study, which found that a supportive social environment that provides opportunities for participation beyond physical accommodations plays a crucial role for youth with physical disabilities (Knibbe et al., 2017). Overall, our findings further support existing environment-based interventions, such as Pathways and Resources for Engagement and Participation (PREP; Law et al., 2016), and emphasize that multiple aspects of the environment can be considered when designing and tailoring interventions aimed at promoting youth and young adults’ participation.

The main category of “utilizing intrinsic factors or qualities” displayed data richness as it encompassed the most distinct generic categories, often observed in one group only. This main category was also recurrent in all settings for both groups. These findings can speak to how children transitioning into adolescence strive to develop autonomy from their parents and seek self-definition (Tsai et al., 2013). This finding suggests that youth can build on the strategies that their parents and caregivers use early on during their childhood to develop self-confidence, autonomy, and a sense of freedom, providing a foundation for developing solutions to participation restrictions and challenges later in life (Kaelin et al., 2021; Killeen et al., 2019). Youth’s need for self-definition and autonomy also appeared in the generic category of “making choices,” mainly used to promote participation at home, community, and the workplace. The notion of “making choices,” speaking to preferences, is defined as the opportunity to choose and to be able to undertake activities that are meaningful or valued as described in the family of Participation-Related Constructs framework (Imms et al., 2017). This framework is used to comprehensively capture the construct of participation. The importance of personal choice was also demonstrated among adolescents with CP to shape and enrich their social participation (Stewart et al., 2012). Similarly, parents listed “respecting youth choices, interests and preferences” as a main strategy to promote participation among youth with physical disabilities (Killeen et al., 2019). Our findings, combined with those from previous studies (Killeen et al., 2019; Stewart et al., 2012), emphasize the importance of enabling young individuals’ participation in meaningful occupations by providing them with opportunities to engage in decision-making and, consequently, using the client-centered approach that is at the core of occupational therapy interventions.

Another generic category underpinning “utilizing intrinsic factors or qualities” speaks to advocacy. Although young people with physical disabilities reported using advocacy-related strategies in the school and workplace settings (especially for accommodations and services), they did not employ such strategies in the community. This could be explained by the characteristics of the community setting (often more complex and less structured), which may limit successful implementation of strategies (Kaelin et al., 2024). Considering that young people with physical disabilities experience participation restrictions (Guyard et al., 2024) and that few policies exist to support participation (Shikako-Thomas & Law, 2015), it is fundamental that occupational therapists work collaboratively with young people with physical disabilities to build on their understanding of the scope and importance of advocacy-related participation strategies. In consequence, this may empower young people with physical disabilities to become agents of change and advocate for themselves to improve programs and services in the community setting. It is noteworthy that even studies about caregiver strategies, such as Kaelin et al. (2021), predominantly discuss strategies at the micro or individual level, focusing on modifying the child’s environment or context (e.g., “put away plastic tableware in easily accessible cabinet”). Recognizing the scarcity of advocacy-related strategies among both young people and caregivers emphasizes the need to address participation restrictions in the community or at the macro level. Occupational therapists can further educate and empower young people and caregivers to advocate for inclusive opportunities and policies to facilitate transitioning to community life.

The strategies used in the school/educational setting displayed the largest discrepancy and most unique responses between the two groups. Many strategies employed by young people with disabilities pertained to benefiting from an accommodative learning environment (e.g., extended deadlines for assignments and exams, access to a specialized education resource teacher). Similarly, one of the most prominent strategies used by parents in the school setting was to modify the institutional environment by advocating to the school teachers, principals, and school boards for specific services and necessary equipment (i.e., walkers, elevators, and lockers for medicine), being active, such as planning field trips and volunteering in class, and collaborating with the school staff to stay informed on school events (Killeen et al., 2019). This could indicate that strategies employed by parents and caregivers have a long-term effect on youth and encourage them to employ similar strategies as they transition to adulthood.

Another meaningful type of strategy, particularly from young people with disabilities, was “analyzing the situation” observed in three of the four settings. In the workplace, this involved many factors for sustained engagement, such as timing and intensity of the activity, scheduling rest breaks throughout workdays, and setting limits on work hours—indicating an important level of self-awareness among this group. These strategies also considered the accessibility of an activity and planning according to pacing, weather conditions, and parking within the community. Similarly, Golos et al. (2023) revealed that strategies focused on ways to engage in activities while considering accessibility, and adapting the environment and activities, implemented by the occupational therapist through PREP intervention, improved participation of youth with disabilities within their community. As in Ding et al.’s (2024) work, our findings further emphasize the complexity of participation for transition-age individuals with disabilities. Findings also stress the need to consider a range of factors, including assessing an activity and its context to adequately respond to the necessary demands. Developing the analytical skills of young people and building strategies to analyze situations are broad and multifaceted ways to promote participation that can be further developed in therapy.

An interesting observation was that young individuals with disabilities did not consider working on improving ICF body functions, structures, or impairments (e.g., doing upper body exercises) as a strategy to promote participation in the home, school/educational, and community settings. This coincides with a previous study examining parents’ strategies to promote participation of youth with physical disabilities (Killeen et al., 2019). It is plausible that modifying the environment is perceived as more applicable and practical compared with addressing body functions. This observation is consistent with our study findings, wherein participants with physical disabilities placed more emphasis on environment-related strategies. Further research is needed to examine this assumption.

Some unique findings with respect to the school, where most differences were observed, are worth reflecting on. To illustrate, young individuals without disabilities employed more strategies in relation to engaging in school activities with peers than young people with disabilities. Similar studies have indicated comparable results where the participation of children and youth with disabilities in school activities and their interaction with peers were significantly lower. Relationships with peers were less frequently reported as a support (Coster et al., 2013). This can redirect both professionals’ and youth’s attention to the social context when co-devising solution-based strategies for promoting participation in school.

### Limitations and Future Directions

The generalizability of study findings should be done with caution. While groups were matched by sex and age, a convenient sampling method was used from mainly one geographical location. Consequently, the study may not fully capture the diversity of experiences among young people with and without disabilities across different regions or demographic profiles, with the potential for selection bias. Individuals with cognitive impairments and intellectual disabilities were also excluded from the sample. Moreover, responses were collected during the COVID-19 pandemic, a particularly challenging period for occupational engagement and participation. This adds another layer of complexity to identifying participation strategies. Furthermore, some participants’ strategies were described very briefly in the Y-PEM and this may have limited interpretation. Future studies should use the Y-PEM alongside semi-structured interviews with the participants to clarify their written responses. Finally, this study exclusively focused on identifying strategies employed by the participants and did not assess the effectiveness of these strategies. Future studies may consider asking young individuals to rank their participation strategies in terms of perceived effectiveness.

## Conclusion

This study provides new knowledge on the participation strategies employed by transition-age young people with and without physical disabilities across four settings: home, school/educational, community, and the workplace. Strategies related to optimizing the physical, social, and institutional environment were identified as crucial in all settings, with seven of the 11 identified categories relating to this domain. The main category of utilizing intrinsic factors or qualities highlighted how young people with disabilities proactively shape their participation experiences with advocacy-related strategies. Overall, the findings emphasize the need for tailored client-led interventions that build on both environment-focused strategies and the empowerment of intrinsic qualities that young individuals already use to promote meaningful participation. These findings hold implications for occupational therapy practitioners, educators, policymakers, and young individuals themselves, by improving their understanding of the range of strategies used by this population, guiding them in crafting targeted interventions and creating supportive environments that enhance participation across various settings. Future research could include a broader range of diagnoses, including cognitive disabilities and mental health problems, to shed light on participation strategies among different disability groups. This could lead to more tailored interventions and policies to enable participation for diverse populations.

## Supplemental Material

sj-docx-1-otj-10.1177_15394492241280198 – Supplemental material for Participation Strategies Used by Young People With and Without Physical Disabilities Supplemental material, sj-docx-1-otj-10.1177_15394492241280198 for Participation Strategies Used by Young People With and Without Physical Disabilities by Andrianantenaina Ornella, Baladzhanov Nikol, Germain Lauriane, Schneidman Lyndsay, Shahin Saeideh and Anaby Dana in OTJR: Occupational Therapy Journal of Research
