# Supplementary material for: Participation Strategies Used by Young People With and Without Physical Disabilities
Source: OTJR (Thorofare N J). 2024 Sep 24;45(3):338–49. doi: 10.1177/15394492241280198 (PMC12130587; doi:10.1177/15394492241280198)
Supplement: sj-docx-1-otj-10.1177_15394492241280198 – Supplemental material for Participation Strategies Used by Young People With and Without Physical Disabilities [file sj-docx-1-otj-10.1177_15394492241280198.docx]

**Supplementary Table 1.**

***Sample Characteristics***

| **Total sample** | **Total Sample** | **With disability (n=52)** | **Without disability (n=54)** |
| --- | --- | --- | --- |
| **Total n** | 106 | 52 (49.1%) | 54 (50.9%) |
| **Age (y), mean (SD)** | 22.7 (4.2) | 22.9 (4.2%) | 22.7 (4.3%) |
| 12–24 | 67 (63.2%) | 33 (63.5%) | 34 (63.0%) |
| 25-30 | 39 (36.8%) | 19 (36.5%) | 20 (37.0%) |
| **Sex** |  |  |  |
| Male | 46 (43.4%) | 22 (42.3%) | 24 (44.4%) |
| Female | 60 (56.6%) | 30 (57.7%) | 30 (55.6%) |
| **Household income** |  |  |  |
| Below $60,000 | 40 (37.7%) | 25 (48.1%) | 15 (27.8%) |
| About $60,000 | 19 (17.9%) | 12 (23.1%) | 7 (13.0%) |
| Above $60,000 | 46 (43.4%) | 14 (26.9%) | 32 (59.3%) |
| Missing | 1 (0.9%) | 1 (1.9%) | 0 (0%) |
| **Type of community** |  |  |  |
| Major urban | 68 (64.2%) | 31 (59.6%) | 37 (68.5%) |
| Suburban | 27 (25.5%) | 12 (23.1%) | 15 (27.8%) |
| Small town | 8 (7.5%) | 6 (11.5%) | 2 (3.7%) |
| Rural | 2 (1.9%) | 2 (3.8%) | 0 (0%) |
| Missing | 1 (0.9%) | 1 (1.9%) | 0 (%) |
| **Language spoken at home** |  |  |  |
| English | 50 (47.2%) | 28 (53.8%) | 22 (40.7%) |
| French | 25 (23.6%) | 21 (40.4%) | 4 (7.4%) |
| Other | 31 (29.2%) | 3 (5.8%) | 28 (51.9%) |
| **Living with** |  |  |  |
| Mother | 77 (72.6%) | 33 (63.5%) | 44 (81.5%) |
| Father | 55 (51.9%) | 21 (40.4%) | 34 (63.0%) |
| Male legal guardian | 1 (0.9%) | 1 (1.9%) | 0 (0%) |
| Female legal guardian | 1 (0.9%) | 0 (0%) | 1 (1.9%) |
| Alone | 21 (19.8%) | 13 (25.0%) | 8 (14.8%) |
| Other | 20 (18.9%) | 7 (13.5%) | 13 (24.1%) |
| **Highest education level** |  |  |  |
| High school or less | 34 (32.1%) | 21 (40.4%) | 13 (24.1%) |
| Some college or university or technical training (at least one year) | 18 (17.0%) | 8 (15.4%) | 10 (18.5%) |
| Graduated college/University | 42 (39.6%) | 18 (34.6%) | 24 (44.4%) |
| Graduate degree | 10 (9.4%) | 3 (5.8%) | 7 (13.0%) |
| Vocational training/Diploma | 2 (1.9%) | 2 (3.8%) | 0 (0%) |
| **Activities engaged in during the week** |  |  |  |
| Working full time | 32 (30.2%) | 12 (23.1%) | 20 (37.0%) |
| Working part-time/ seasonal | 30 (28.3%) | 11 (21.2%) | 19 (35.2%) |
| Looking for work | 13 (12.3%) | 6 (11.5%) | 7 (13.0%) |
| Going to school | 58 (54.7%) | 23 (44.2%) | 35 (64.8%) |
| Recovering from illness | 9 (8.5%) | 8 (15.4%) | 1 (1.9%) |
| Volunteering | 14 (13.2%) | 7 (13.5%) | 7 (13.0%) |
| Other | 5 (0.5%) | 2 (3.8%) | 3 (5.6%) |
